# Glucose confers protection to *Escherichia coli* against contact killing by *Vibrio cholerae*

**DOI:** 10.1038/s41598-021-81813-4

**Published:** 2021-02-03

**Authors:** Cristian V. Crisan, Holly L. Nichols, Sophia Wiesenfeld, Gabi Steinbach, Peter J. Yunker, Brian K. Hammer

**Affiliations:** 1 School of Biological Sciences, Georgia Institute of Technology, Atlanta, GA USA; 2 Parker H. Petit Institute for Bioengineering & Bioscience, Georgia Institute of Technology, Atlanta, GA USA; 3 Center for Microbial Dynamics and Infection, Georgia Institute of Technology, Atlanta, GA USA; 4 School of Physics, Georgia Institute of Technology, Atlanta, GA USA

**Keywords:** Bacteriology, Bacterial genetics, Microbial ecology

## Abstract

Evolutionary arms races are broadly prevalent among organisms including bacteria, which have evolved defensive strategies against various attackers. A common microbial aggression mechanism is the type VI secretion system (T6SS), a contact-dependent bacterial weapon used to deliver toxic effector proteins into adjacent target cells. Sibling cells constitutively express immunity proteins that neutralize effectors. However, less is known about factors that protect non-sibling bacteria from T6SS attacks independently of cognate immunity proteins. In this study, we observe that human *Escherichia coli* commensal strains sensitive to T6SS attacks from *Vibrio cholerae* are protected when co-cultured with glucose. We confirm that glucose does not impair *V. cholerae* T6SS activity. Instead, we find that cells lacking the cAMP receptor protein (CRP), which regulates expression of hundreds of genes in response to glucose, survive significantly better against *V. cholerae* T6SS attacks even in the absence of glucose. Finally, we show that the glucose-mediated T6SS protection varies with different targets and killers. Our findings highlight the first example of an extracellular small molecule modulating a genetically controlled response for protection against T6SS attacks. This discovery may have major implications for microbial interactions during pathogen-host colonization and survival of bacteria in environmental communities.

## Introduction

*Vibrio cholerae* is the waterborne enteric pathogen that causes serious, often fatal cholera diarrheal disease when ingested by humans. This ubiquitous microbe is found in dense polymicrobial marine communities on chitinous surfaces and in animal reservoirs like fish, zooplankton or insects^1–4^. To compete with other cells in densely populated microbial environments, *V. cholerae* employs a harpoon-like type VI secretion system (T6SS)^5–8^. The *V. cholerae* T6SS punctures adjacent cells and delivers toxic effector proteins that disrupt lipid membranes and cell walls of the bacterial envelope^9–11^. In animal models, *V. cholerae* uses T6SS effectors to eliminate “target” commensal bacteria like *Escherichia coli* and *Aeromonas veronii*^12,13^. *V. cholerae* strains with an active T6SS exhibit increased pathogenicity and cause severe cholera-like symptoms in these animal model systems^12–14^, suggesting the apparatus plays important roles in enhancing the ability of *V. cholerae* to colonize environmental habitats and infect hosts. While many studies have investigated T6SS offensive abilities, less is known about mechanisms of protection against T6SS aggression.

Bacterial “killer” cells with active T6SSs constitutively express immunity proteins that recognize and neutralize toxic effectors^10,11,15^. The survival of *V. cholerae* target cells devoid of immunity proteins is modestly increased by enhanced production of secreted exopolysaccharides, presumably by creating a physical barrier between the killer and target strains^16,17^. Modifications in the cell wall composition of *Acinetobacter baumannii* confer protection against T6SS attacks^18^. Furthermore, envelope stress response systems play important roles in the survival of target cells against T6SS attacks^17,19–22^.

Environmental conditions and small molecules can modulate T6SS-mediated antagonism by *Vibrios*, but studies of external conditions that trigger target cells to overcome attacks are lacking^21,23,24^. Here we report that exogenous glucose protects human commensal *E. coli* strains from T6SS-mediated aggression by *V. cholerae*. Protection varies with the target and killer strains tested and is mediated by the target cells’ cyclic adenosine monophosphate (cAMP) receptor protein (CRP) glucose response regulator. Since glucose decreases pathogen colonization of the intestinal tract and alters interactions between *V. cholerae* and *E. coli* cells in hosts^25,26^, induced protection from T6SS attacks by metabolites may be common in microbial communities of environmental and human health importance.

## Results

### Exogenous glucose protects *E. coli* target cells against T6SS attacks

**Figure 1:**
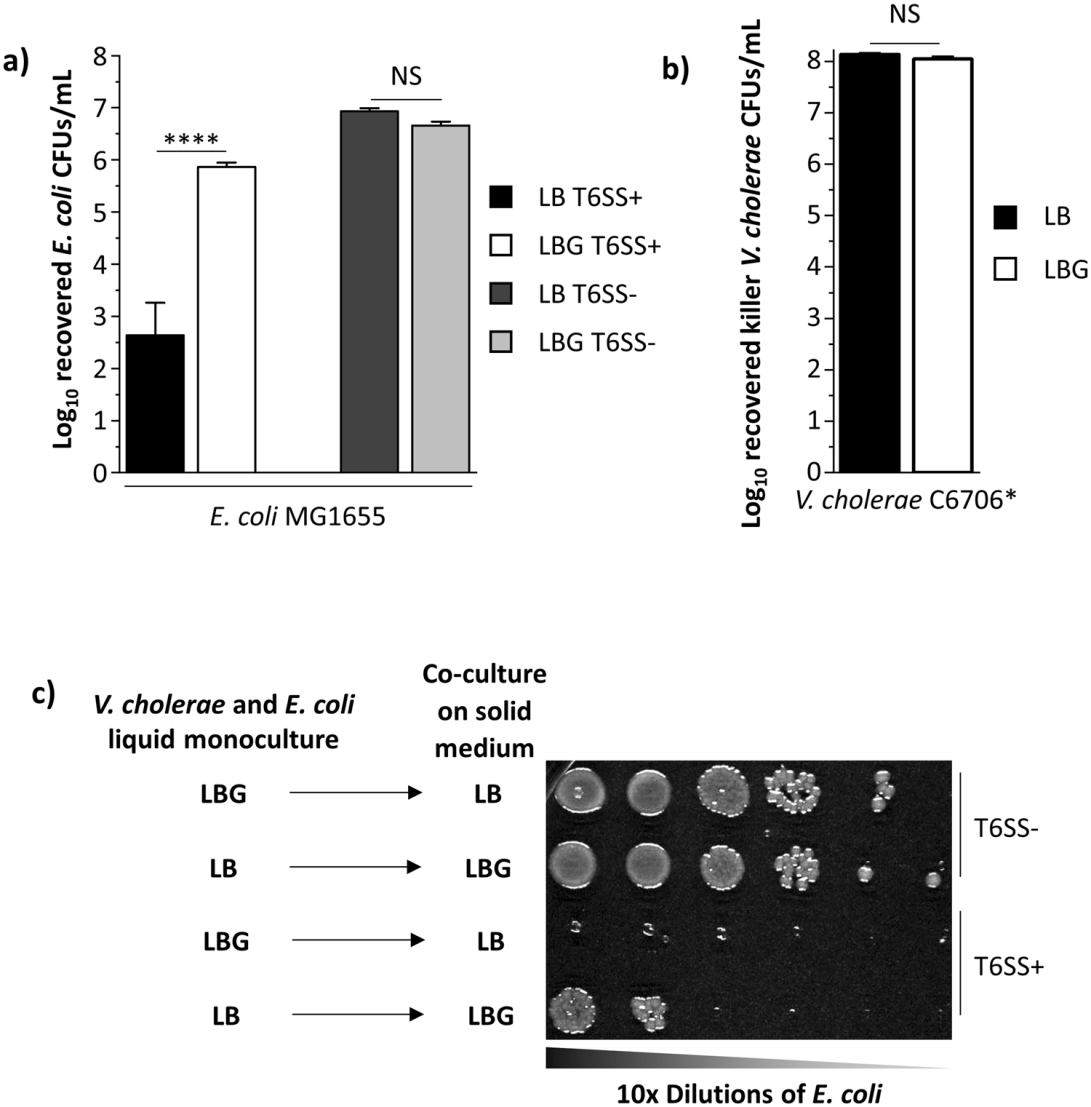
*E. coli* cells are protected against *V. cholerae* T6SS attacks when co-cultured in the presence of glucose. (**a**) *E. coli* MG1655 cells were co-cultured for 3 h with either killer T6SS+ or defective T6SS- *V. cholerae* C6706* cells on LB or LBG plates. The number of recovered chloramphenicol-resistant *E. coli* cells was determined by counts of colony forming units (CFUs) after co-culture mixtures were diluted and spread on chloramphenicol plates. A two-way ANOVA with a Bonferroni post hoc test was performed to determine significance. A minimum of 3 independent replicates were analyzed. (**b**) The same competition assay was performed as described above. However, the number of recovered killer *V. cholerae* C6706* cells was determined instead. A Welch’s t-test was performed to determine significance. A minimum of 3 independent replicates were analyzed. (**c**) *V. cholerae* C6706* cells grown overnight in liquid LB or LBG were co-cultured with *E. coli* on solid LBG or LB media, respectively, followed by dilution and plating as described in the “Methods” section. ****p < 0.0001, *NS* not significant.

When co-cultured on rich LB medium, *V. cholerae* pandemic isolate C6706 constitutively expressing the QstR master gene regulator (denoted here as C6706*) kills *E. coli* MG1655 target cells efficiently (Fig. 1a)^27–30^. By contrast, a T6SS- *V. cholerae* C6706* strain with a deletion in the gene encoding the essential T6SS protein VasK is unable to eliminate target cells (Fig. 1a)^31^. When *V. cholerae* C6706* and *E. coli* MG1655 cells are co-cultured on LB medium containing 0.4% glucose (LBG), reflecting physiological levels, the number of recovered *E. coli* cells is significantly increased (~ 1000-fold) compared to co-cultures on LB with no added glucose (Fig. 1a)^32,33^. By contrast, the number of recovered *V. cholerae* killer cells is unaltered when co-cultured for three hours with *E. coli* MG1655 on LB and LBG (Fig. 1b).

To determine whether *E. coli* cells require active glucose induction to withstand T6SS attacks, we incubated *V. cholerae* C6706* and *E. coli* MG1655 in liquid LBG or LB and then co-cultured the strains on either solid LB or solid LBG medium, respectively (Fig. 1c). *E. coli* cells overcome T6SS attacks when incubated in liquid LB and co-cultured with *V. cholerae* C6706* on solid LBG but are poorly recovered when incubated in liquid LBG and co-cultured on solid LB (Fig. 1c). We also hypothesized that glucose could allow *E. coli* cells to escape T6SS attacks by replicating faster and evading killer cells^34^. However, when grown individually in monoculture conditions that mimic co-culture assays, recovered *E. coli* and *V. cholerae* cell numbers are unaltered on LBG compared to LB (Supplementary Fig. S1). Thus, *E. coli* cells susceptible to a killer overcome T6SS attacks but do not replicate significantly faster when glucose is present.

### The identities of target and killer strains alter protection against T6SS attacks

**Figure 2:**
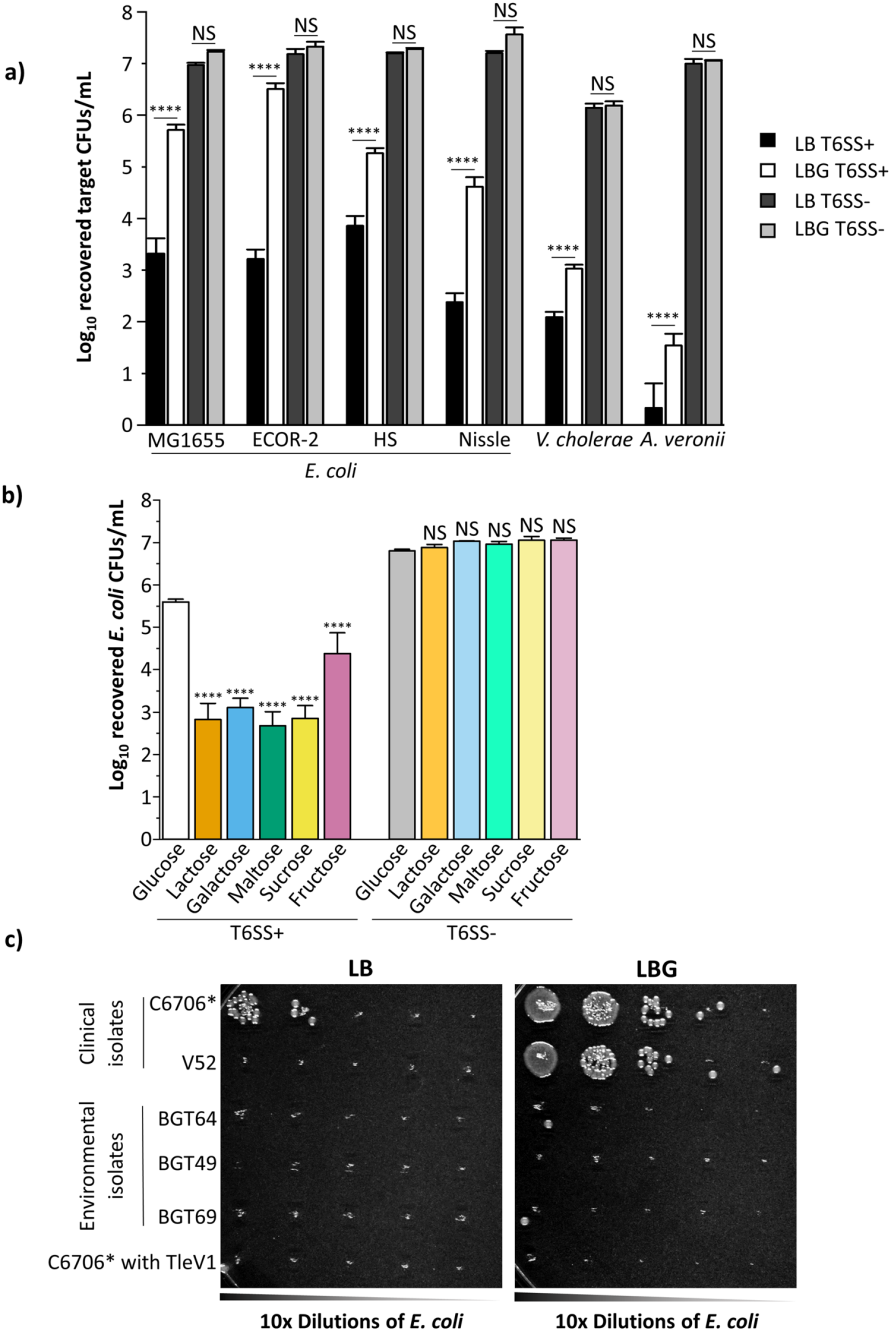
Protection against T6SS attacks depends on the target strain, the killer strain and the sugar substrate. (**a**) Along with MG1655, *E. coli* commensal strains ECOR-2, HS, Nissle, susceptible *V. cholerae* and *A. veronii* target cells were co-cultured with killer *V. cholerae* C6706* or C6706* T6SS- on LB or LBG. A three-way ANOVA with a Bonferroni post hoc test was performed to determine significance. A minimum of 3 independent replicates were analyzed. (**b**) Killer *V. cholerae* C6706* cells were co-cultured with *E. coli* MG1655 cells on LB medium containing 0.4% of the indicated sugar compounds. A two-way ANOVA with a post hoc Tukey HSD test was used to determine significance. A minimum of 3 independent replicates were analyzed. (**c**) Clinical killers *V. cholerae* C6706*, V52 and C6706* with TleV1, as well as environmental strains BGT49, BGT64 and BGT69 were co-cultured with *E. coli* MG1655 cells on LB medium (left) or LBG medium (right) and then diluted and plated as described in the “Methods” section. ****p < 0.0001, *NS* not significant.

To test whether the robust glucose-mediated protection is specific to *E. coli* MG1655, we co-cultured killer *V. cholerae* C6706* with other human *E. coli* commensal strains (Nissle, HS and ECOR-2)*,* as well as fish symbiotic *A. veronii* and susceptible *V. cholerae* that lacks all three immunity proteins for C6706* T6SS effectors^13,35–41^. All tested target strains have increased recovery when glucose is added to the medium (Fig. 2a). Importantly, the recovery difference on LBG compared to LB is greater than one order of magnitude for all the *E. coli* target tests, while *A. veronii* and *V. cholerae* targets are still efficiently eliminated even in the presence of glucose (Fig. 2a). The number of recovered target cells when competed against C6706* T6SS-deficient killers on LB or LBG medium is unchanged (Fig. 2a).

We next investigated whether the protection of *E. coli* against T6SS attacks is specific to glucose. We co-cultured killer C6706* and target *E. coli* MG1655 on LB medium containing different sugars, each at a concentration of 0.4%: fructose and galactose (monosaccharides), as well as sucrose, maltose and lactose (glucose-containing disaccharides). No other sugar permits the recovery observed with glucose, while fructose provides an intermediate level of protection (Fig. 2b, see “Discussion” section). When identical co-culture experiments are conducted using T6SS- defective killer C6706* cells, the number of recovered *E. coli* MG1655 cells is similar for all tested sugars (Fig. 2b).

To determine whether the effects on *E. coli* we observed were generalizable to other killers, we examined T6SS attacks from other *V. cholerae* strains. *V. cholerae* clinical strain V52 constitutively expresses T6SS genes and encodes the same toxins as C6706^11,42,43^. Like C6706*, V52 poorly eliminates *E. coli* MG1655 on LBG (Fig. 2c). Strains BGT49, BGT64 and BGT69 were isolated from environmental, rather than clinical, sources^44,45^. These three strains engage in robust contact killing with effector toxins different from clinical strains and efficiently eliminate *E. coli* despite glucose supplementation (Fig. 2c)^44,45^. We recently reported that an engineered C6706* strain encoding the additional TleV1 effector originally found in strain BGT49 (but not in other strains tested here) kills parental *V. cholerae* C6706 cells lacking the cognate immunity gene^44^. Surprisingly, C6706* with TleV1 can kill *E. coli* even in the presence of glucose (Fig. 2c). These results reveal that glucose protection varies with target cells and with effector toxins deployed by different killers.

### *V. cholerae *can efficiently use its T6SS when co-cultured with *E. coli* cells in the presence of glucose

**Figure 3:**
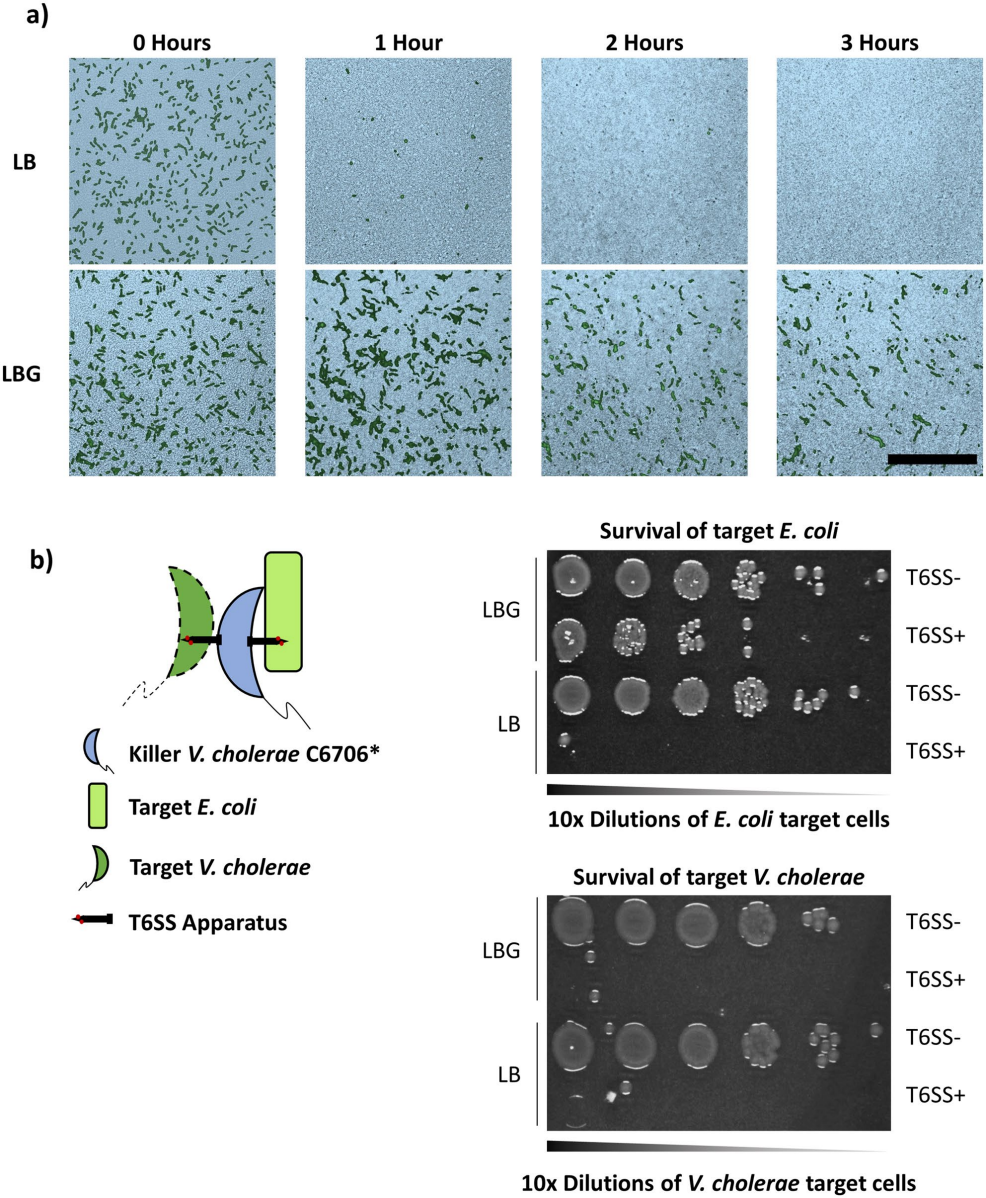
Individual *E. coli* MG1655 cells are protected against *V. cholerae* T6SS attacks but do not impair T6SS killing. (**a**) Fluorescently labeled green *E. coli* MG1655 cells were densely co-cultured with unlabeled *V. cholerae* C6706* on LB (top panels) or LBG (bottom panels) and the same frame was imaged for three hours using confocal microscopy. The pseudocolor images depict the progression of T6SS killing at different time points, showing brightfield microscopy images (bright blue) of the dense biofilm overlaid with the fluorescence signal of *E. coli* cells projected on a plane parallel to the agar substrate. Scale bar is 50 µm. (**b**) Killer *V. cholerae* C6706* or C6706* T6SS- was co-cultured with both *E. coli* MG1655 and susceptible *V. cholerae* target cells, and then diluted and plated as described in the “Methods” section.

To confirm that individual *E. coli* cells are protected from T6SS attacks on LBG, we imaged co-cultures of sfGFP-labelled *E. coli* MG1655 with unlabeled *V. cholerae* C6706* by confocal microscopy. On LB medium, *E. coli* cells are eliminated after 3 h (Fig. 3a, top panels). By contrast, on LBG, multi-cell *E. coli* clusters surrounded by *V. cholerae* cells form and can be observed after 3 h (Fig. 3a, bottom panels). We also examined the membrane integrity of *E. coli* cells when competed against *V. cholerae* on LB or LBG. Propidium iodide (PI) is a DNA-binding dye that exhibits high fluorescence when bound to the DNA of cells with compromised membranes. We observed a PI signal for co-cultures on both LB and LBG (Supplementary Fig. S2). However, on LBG, the PI signal does not overlap with the signal from surviving sfGFP-labelled *E. coli* cells, indicating that the cells’ membranes remain intact (Supplementary Fig. S2).

To determine if *E. coli* can reduce the efficiency of T6SS-mediated killing in the presence of glucose, we performed a polyculture assay in which *V. cholerae* C6706* killer (or C6706* T6SS-) cells were cultured simultaneously with both *E. coli* and susceptible *V. cholerae* that lack immunity proteins for C6706* T6SS effectors and are defective for T6SS killing (Fig. 3b). Under these conditions, *E. coli* cells are protected from T6SS attacks and replicate as expected, while target *V. cholerae* cells are efficiently killed by the C6706* but not by C6706* T6SS- killer (Fig. 3b). Taken together, these results reveal that glucose allows individual *E. coli* cells to survive active T6SS attacks.

### A *crp *disruption protects *E. coli* cells against T6SS attacks even in the absence of glucose

**Figure 4:**
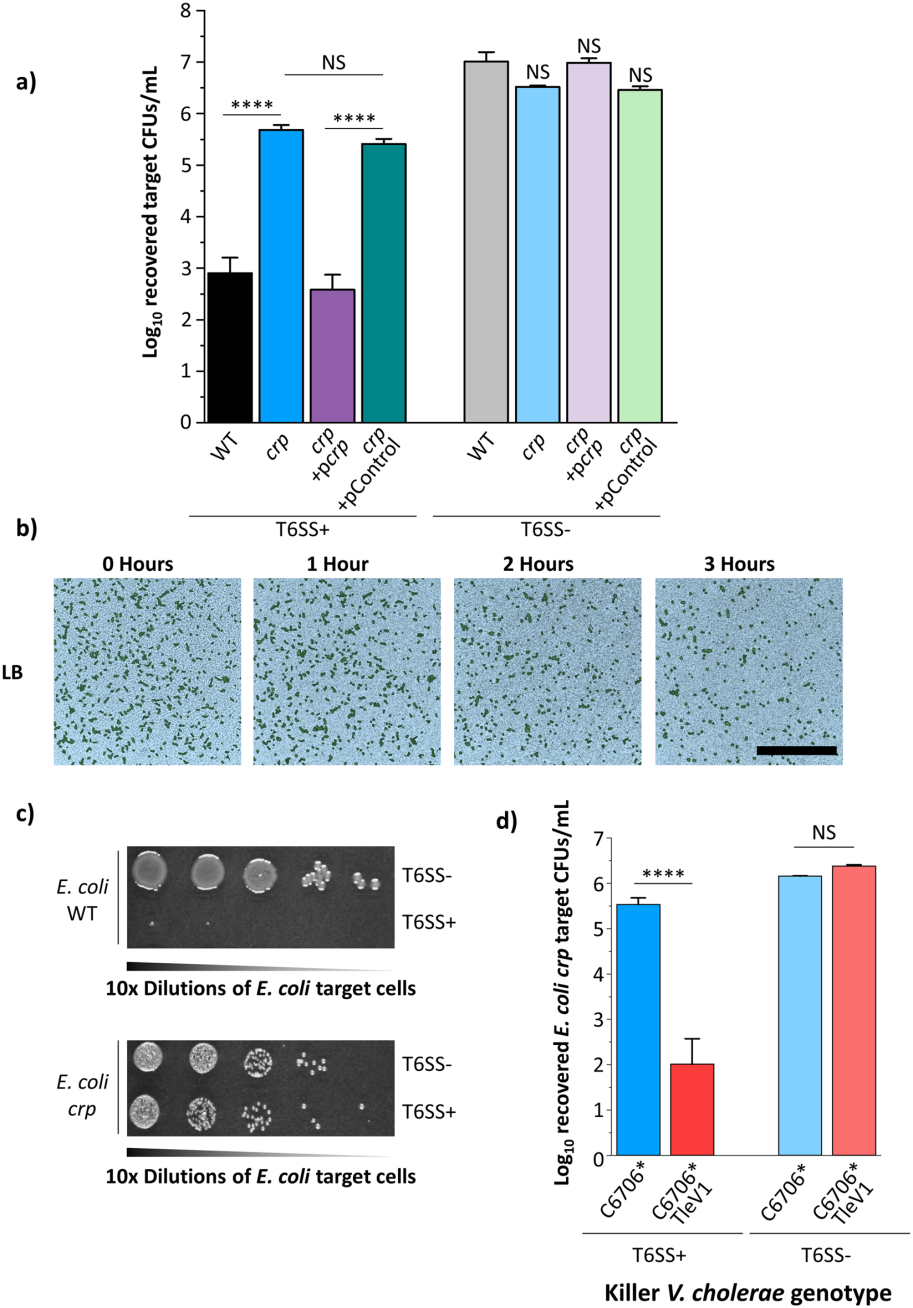
A *crp* disruption confers protection to *E. coli* MG1655 cells in the absence of glucose. (**a**) *E. coli* MG1655 cells with a *crp* disruption harboring the indicated plasmids were co-cultured with *V. cholerae* C6706* (or *V. cholerae* C6706* T6SS-) on LB medium. A two-way ANOVA with a post hoc Bonferroni test was used to determine significance. A minimum of 3 independent replicates were analyzed. (**b**) Fluorescently labeled green *E. coli* MG1655 *crp* cells were densely co-cultured with unlabeled killer *V. cholerae* C6706* on LB and the same frame was imaged for three hours using confocal microscopy. Brightfield images (bright blue pseudocolor) were overlaid with the fluorescence signal of *E. coli* cells projected on a plane parallel to the agar substrate. Scale bar is 50 µm. (**c**) Killer *V. cholerae* C6706* was co-cultured with both wild type *E. coli* MG1655 and *E. coli crp* target cells and then diluted and plated as described in the “Methods” section. (**d**) *V. cholerae* C6706* or *V. cholerae* C6706* with TleV1 (or the T6SS- mutant of each one) were co-cultured with *E. coli* MG1655 *crp* on LB medium. A two-way ANOVA with a post hoc Bonferroni test was used to determine significance. A minimum of 3 independent replicates were analyzed. ****p < 0.0001, *NS* not significant.

We wondered whether a physiological response to glucose levels could mediate the *E. coli* protection. In *E. coli* and other bacteria, low glucose conditions lead to accumulation of intracellular cyclic adenosine monophosphate (cAMP), which binds to and activates the cAMP receptor protein (CRP)^46^. CRP transcriptionally controls expression of hundreds of genes for varied behaviors^46^. Since high glucose conditions are mimicked by a *crp* disruption, we integrated an antibiotic resistance cassette into the *crp* gene on the chromosome of *E. coli* MG1655 (*E. coli crp*). When co-cultured with *V. cholerae* C6706* on LB lacking glucose, the number of recovered *E. coli crp* cells is comparable to that of wild type *E. coli* cells co-cultured with *V. cholerae* on LBG (Figs. 1a, 4a). Complementation with a plasmid expressing *crp* restores *E. coli* susceptibility to T6SS attacks (Fig. 4a). Using confocal microscopy, we confirmed that the *crp* disruption confers protection against T6SS attack to *E. coli* cells even in the absence of glucose (Fig. 4b). After 3 h, distinct clusters of sfGFP-labelled *E. coli crp* cells are visible when co-cultured with *V. cholerae* C6706* on LB, similar to wild type *E. coli* on LBG (Figs. 3a, 4b)*.* We also observed that in a polyculture assay where *V. cholerae* C6706* killer cells are cultured with both the MG1655 *crp* disruption mutant and wild type MG1655, the MG1655 *crp* disruption mutant strain is protected against T6SS attacks while wild type MG1655 is successfully eliminated (Fig. 4c). Consistent with results on LBG (Fig. 2c), *V. cholerae* C6706* (but not C6706* T6SS-) with TleV1 efficiently kills *E. coli crp* on LB lacking glucose (Fig. 4d). These findings reveal that both exogenous glucose and a loss of CRP confer protection to attacks by some T6SS effectors.

## Discussion

Bacteria living in natural complex polymicrobial communities with limited resources are engaged in evolutionary arms races with other prokaryotes, eukaryotes and viruses^47–49^. Competition mechanisms include secretion of antibiotics, lysis by phages, and contact-dependent killing via the T6SS^50,51^. Antibiotic resistance and anti-CRISPR systems have emerged in response to these activities^50–52^. In addition to immunity proteins that neutralize effectors, it is becoming more apparent that arms races between T6SS killers and target bacterial cells have also led to the emergence of diverse defensive mechanisms we are beginning to identify^17,34,53–55^.

Cells lacking immunity proteins for cognate effectors are typically susceptible to T6SS attacks, but physical separation, stress response regulators and polysaccharide secretion contribute to protection from T6SS attacks^16,17,20–22,34,53^. We find that *E. coli* MG1655 overexpressing the *rcsA* gene for colanic acid production from a plasmid, though mucoid in appearance, only have a modest improvement in survival against *V. cholerae* C6706* T6SS killing on LB (Supplementary Fig. S3)^17,56^. Since this increase is several orders of magnitude lower than the protection induced by glucose, production of colanic acid is unlikely to be the primary factor responsible for the glucose-mediated protection in *E. coli* against *V. cholerae* T6SS attacks observed here (Supplementary Fig. S3, Fig. 1). Furthermore, because *E. coli* growth rates on LB or LBG are not significantly different during a 3 h monoculture growth assay (Supplementary Fig. S1), the glucose-mediated protection is not simply a consequence of initial large target cell domains that outnumber *V. cholerae* cells as reported previously^34^. This conclusion is also supported by our confocal microscopy results, where we observe persistence and then replication of single *E. coli* cells outnumbered and surrounded by *V. cholerae* cells (Fig. 3a).

Previously, glucose has been shown to alter interactions between pandemic *V. cholerae* strains and other bacteria^25,26^. In zebrafish hosts, *E. coli* cells inhibit growth of *V. cholerae* by secreting acids in the presence of glucose^26^. Although acid-induced growth inhibition of *V. cholerae* can occur after extended co-culture with an acid-producing bacterial species, we observe that the number of recovered *V. cholerae* cells is unaltered when co-cultured for 3 h with *E. coli* on LB medium with or without glucose (Fig. 1b)^25,26^. Acid stress response pathways contribute to protection of *E. coli* cells against *V. cholerae* T6SS attacks^17,22^. Consistent with this observation, we also observe that buffering LBG media at a near-neutral pH of 7.4 enhances killing by approximately tenfold (Supplementary Fig. S4). However, this is again insufficient to explain the 1000-fold protection imparted by glucose (Supplementary Fig. S4, Fig. 1a). It remains possible that acidic conditions created by secreted acetate during aerobic growth on LBG medium could trigger stress response pathways in *E. coli* cells, but these effects alone likely play a modest role in the protection from T6SS attacks observed here.

**Figure 5:**
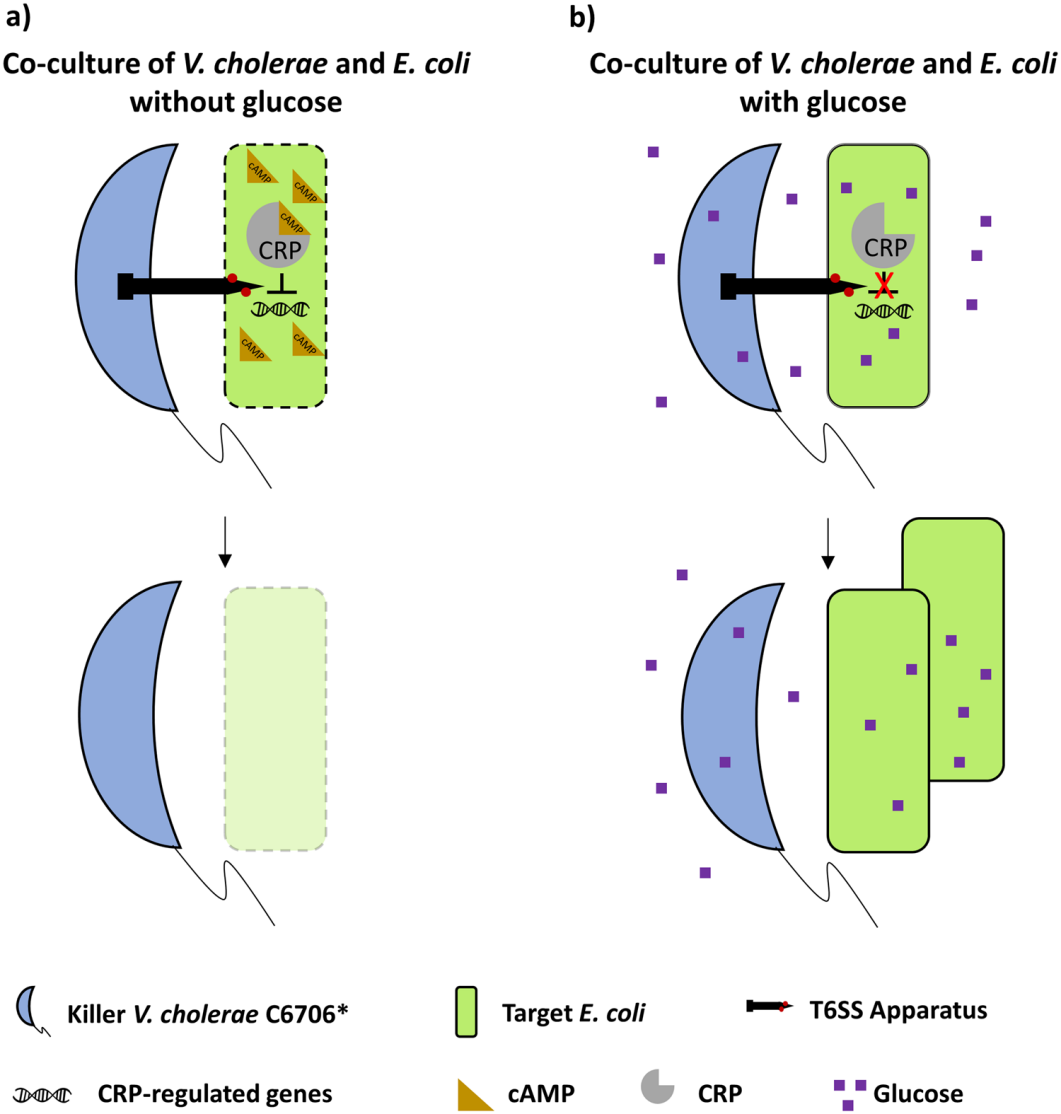
Model depicting the proposed *E. coli* glucose-mediated protection mechanism against the *V. cholerae* C6706* T6SS. (**a**) When *E. coli* cells are grown on rich media in the absence of glucose, CRP is bound by cAMP and represses (directly or indirectly) an unknown gene (or genes). Repression of those genes results in higher susceptibility to T6SS attacks. (**b**) When *E. coli* cells are grown on rich media in the presence of glucose, CRP is not bound by cAMP and cannot repress the genes it controls under low glucose conditions. As a result, *E. coli* cells have increased protection against T6SS attack.

The addition of other sugars to LB media results in significantly fewer recovered *E. coli* cells compared to glucose, with fructose conferring an intermediate level of protection (Fig. 2b). Both fructose and glucose transporters have been shown to alter cAMP levels and CRP activity, which could explain why both sugars confer *E. coli* cells protection (Fig. 2b)^46,57–60^. We find that an *E. coli crp* disruption mutant is protected from *V. cholerae* C6706* attacks on LB (without glucose induction) in a similar manner to wild type *E. coli* on LBG (Fig. 4a). This indicates that CRP likely directly or indirectly represses a gene or genes that confers *E. coli* MG1655 protection against T6SS attacks (Fig. 5). CRP controls expression of genes involved in stress-response pathways^61–63^. Furthermore, CRP represses expression of antibiotic resistance genes in *E. coli*^64^*.* Since envelope damage, acidic stress and osmotic stress response pathways have been recently shown to increase protection against T6SS attacks, we hypothesize that CRP could also repress one or more stress response genes that confer protection against the T6SS^17,20,22^.

All tested human commensal *E. coli* strains survive T6SS attacks significantly better on LBG medium compared to LB, suggesting the glucose-mediated protection is widespread among diverse *E. coli* strains (Fig. 3a)^35,37–39,41^. Even though susceptible target *V. cholerae* and *A. veronii* strains display an increase in the number of recovered cells when the LB medium is supplemented with glucose, these target cells are efficiently eliminated, and the protection is much reduced compared to *E. coli* strains (Fig. 2a). The glucose-mediated protection also depends on the identity of the killer strain. Clinical strain V52 also kills *E. coli* MG1655 to a lesser degree with glucose present, similar to pandemic C6706*, although we observed more variation between replicates in the number of recovered *E. coli* cells when co-cultured with V52 on LBG (Fig. 2c, data not shown). Environmental *V. cholerae* strains, which encode different effectors than V52 or C6706, efficiently kill *E. coli* even in the presence of glucose (Fig. 2c)^44^. These results suggest that the toxicity of T6SS effectors may be differentially affected by glucose-mediated changes in *E. coli*.

The observation that *V. cholerae* C6706* killer cells carrying an additional, non-native effector can bypass the protection conferred by glucose and effectively kill *E. coli* also supports our conclusion that glucose does not prevent effector delivery by the killer into *E. coli* cells (Fig. 2c). These results suggest that the acquisition of diverse toxins might be an evolutionary adaptive strategy for bypassing protective mechanisms developed in potential target strains under fluctuating external conditions^28,36,44,65^. It is possible that the ability of certain killer strains to metabolize glucose might contribute to glucose-induced T6SS protection in some killer-target interactions. However, since CRP controls stress response pathways which might increase protection of *E. coli* against T6SS attacks, we favor a model that CRP-induced changes in the *E. coli* cell envelope may affect the toxicity of some *V. cholerae* T6SS effectors^17,22,62–64^.

To our knowledge, this study highlights the first demonstration that an external molecule can induce a genetic response that increases protection to T6SS attacks in target cells. Zhao et al. have shown that T6SS-mediated killing of *E. coli* cells by *V. cholerae* enhances cholera symptoms in mouse model systems^12^. We speculate that glucose or other external metabolites in the digestive tract could contribute to the neutralization by commensals of T6SS attacks from members of the gut microbiota or foreign pathogens^66^. Future experiments will identify the roles played by stress response pathways in the glucose-mediated protection to T6SS, determine the role of this protection in vivo, and study the effects glucose has on other killer and target bacterial species, including members of the human gut microbiome.

## Methods

### *V. cholerae* and *E. coli* genetic mutations

All *V. cholerae* mutant strains were made as previously described using the pKAS allelic exchange methods^67^. The *E. coli crp* mutant strain was made using the Lambda Red system^68,69^. The *E. coli* strain expressing sfGFP was made using an integration event from a pKAS construct. *E. coli* strains Nissle, HS and ECOR-2, as well as *A. veronii* and *V. cholerae* target cells harbored the pSLS3 plasmid to confer chloramphenicol resistance. Restriction enzymes, polymerases and Gibson mix reagents were used according to the manufacturer’s instructions (Promega and New England Biolabs). Plasmids were verified by PCR and Sanger sequencing (Eurofins). All strains and plasmids are described in Supplementary Table S1.

### Bacterial co-culture assays

Bacterial cultures were grown in liquid LB or LB + glucose 0.4% (LBG) media with shaking at 37 °C overnight. Cultures were back-diluted, incubated at 37 °C with shaking for 3 h in the same conditions as the overnight cultures and the absorbance for each sample was set to an OD_600_ of 1. Strains harboring plasmids were grown in overnight liquid media with the respective antibiotics required to maintain plasmids and were washed three times with fresh media before co-cultured. Killer and target strains were mixed at a 10:1 (killer:target) ratio and 50 µL of the mixture was spotted on a filter paper with a 0.22 µm pore size. For polyculture assays, strains were mixed at a ratio of 10:1:1 (killer:target:target). The filter was placed on LB or LBG agar media and incubated at 37 °C for 3 h. Filters were vortexed in 5 mL of sterile LB medium for 30 s and 100 µL (or 3 μL for spot plating) of serial dilutions were spread on chloramphenicol plates to select for target cells. At least 3 independent replicates were used for statistical comparisons.

Modified co-culture assays were performed as described above for different conditions. Different sugars (fructose, sucrose, lactose, galactose and maltose) were added to LB to a final concentration of 0.4%. Overnight cultures, back-diluted cultures and co-culture experiments were performed on medium containing the respective sugar. Co-culture assays using buffered media were performed on LBG medium adjusted to a pH of 7.4 and buffered with 40 mM MOPS. Experiments were also conducted with buffered LBG medium containing 40 mM HEPES or 100 mM phosphate buffers and similar results were obtained. Overnight cultures, back-diluted cultures and co-culture experiments were performed on pH 7.4 buffered LBG medium. Strains expressing *rcsA* and *crp* were induced with 100 µM IPTG during overnight cultures and co-cultures with killer cells.

### Confocal microscopy

Confocal microscopy experiments were performed as described previously^44^. Briefly, overnight cultures were back-diluted 1:100 for 3 h in the same media as the overnights and the concentration of each sample was set to an OD_600_ of 10. A 4 µL droplet aliquot of propidium iodide (100 µg/mL) was added to an LB or LBG agar pad on a glass slide and allowed to dry. Next, a 1 µL aliquot of a 10:1 killer:target cell mixture was spotted onto the propidium iodide drop on the agar pad. Cells were imaged at 96–100% humidity and 37 °C using a Nikon A1R confocal microscope. At different time points, a three-dimensional stack of the biofilm was recorded with lateral dimensions of 118 µm × 118 µm and a vertical step size of 1 µm. Fluorescence images were obtained by projecting all slices of the three-dimensional stack onto one plane parallel to the agar substrate. Those projected fluorescence images were overlaid onto a cross-sectional bright-field image of the biofilm. A Perfect Focus System with a 40 × objective (Plan Fluor ELWD 40 × DIC M N1) was used to stabilize the focus in the plane of the colony growth. Images were processed using ImageJ.

## Supplementary Information

Supplementary Table S1.

Supplementary Figures.
