# Supplementary material for: Glucose confers protection to Escherichia coli against contact killing by Vibrio cholerae
Source: Sci Rep. 2021 Feb 3;11:2935. doi: 10.1038/s41598-021-81813-4 (PMC7858629; doi:10.1038/s41598-021-81813-4)
Supplement: Supplementary file 1 — Supplementary Table S1. [file 41598_2021_81813_MOESM1_ESM.pdf]

Table S1: *E. coli* and *V. cholerae* strains and plasmids used in this study.

Bacterial Strains

Strain Number	Strain Background	Genotype	Plasmid	Reference
**CC151**	NRD204<br>*E. coli* MG1655	*araBAD::cat*		De Lay and Cronan. J. Biol. Chem. 2007
**CC130**	*E. coli* Nissle	WT	pSLS3	Sonnenborn. FEMS Microbiol Lett. 2016.<br>This study
**CC142**	*E. coli* ECOR-2	WT	pSLS3	Ochman and Selander. 1984. J. Bacteriol.<br>This study
**CC143**	*E. coli* HS	WT	pSLS3	Levine et al. Lancet. 1978.<br>This study
**CC116**	*E. coli* MG1655	*araBAD::cat*<br>*lacZ::pKAS-sfGFP*		This study
**CC147**	*E. coli* MG1655	*araBAD::cat*	pUC18-*rcsA*	This study
**SSW11**	*E. coli* MG1655	*araBAD::cat*	pUC18	This study
**CC132**	*E. coli* MG1655	*araBAD::cat*<br>*crp::specR*		This study
**CC133**	*E. coli* MG1655	*araBAD::cat*<br>*crp::specR*	pEVS143-*crp*	This study
**CC140**	*E. coli* MG1655	*araBAD::cat*<br>*crp::specR*	pEVS143	This study
**SSW17**	*E. coli* MG1655	*araBAD::cat*<br>*crp::specR*<br>*lacZ::pKAS-sfGFP*		This study
**JT101**	*V. cholerae*<br>C6706* T6SS+	*ptac-qstR*		Thomas et al. mBio. 2017, This study
**JT932**	*V. cholerae*<br>C6706* T6SS-	*ptac-qstR*<br>Δ*vasK*		Thomas et al. mBio. 2017, This study
**CC144**	*V. cholerae*<br>C6706 target	Δ*vasK lacZ::specR*<br>Δ*tsiV1* Δ*tsiV2* Δ*tsiV3*	pSLS3	This study
**SW491**	*V. cholerae* V52	WT		Pukatzki et al. PNAS. 2006
**BGT49**	*V. cholerae* VC56	WT		Bernardy et al. Appl. Env. Micro. 2016.
**BGT64**	*V. cholerae*<br>692-79	WT		Bernardy et al. Appl. Env. Micro. 2016.
**BGT69**	*V. cholerae*<br>3223-74	WT		Bernardy et al. Appl. Env. Micro. 2016.

**KW25**	*V. cholerae C6706** T6SS+	*ptac-qstR lacZ::aux 5a*		Crisan et al. Genome Bio. 2019
**CC145**	*Aeromonas veronii*	WT	pSLS3	This study
**JT821**	*V. cholerae* C6706	Δ*vasK lacZ*::*specR* Δ*tsiV1* Δ*tsiV2* Δ*tsiV3*		This study
**CC159**	*E. coli* MG1655	WT	pEVS143	This study
**KW26**	*V. cholerae C6706** T6SS+ Δ*vasK*	*ptac-qstR* Δ*vasK lacZ::aux 5a*		Crisan et al. Genome Bio. 2019

Plasmids

Plasmid	Features	Antibiotic Resistance	Reference
**pSLS3**		Chloramphenicol	Tu and Bassler. Genes Dev. 2007
**pUC18**		Ampicillin	Norrander et al. Gene. 1983.
**pUC18-*rcsA***	*ptac-rcsA*	Ampicillin	This study
**pEVS143**		Kanamycin	Dunn et al. Appl. Env. Micro. 2006.
**pEVS143-*crp***	*ptac-crp*	Kanamycin	This study
