# Supplementary material for: Glucose confers protection to Escherichia coli against contact killing by Vibrio cholerae
Source: Sci Rep. 2021 Feb 3;11:2935. doi: 10.1038/s41598-021-81813-4 (PMC7858629; doi:10.1038/s41598-021-81813-4)
Supplement: Supplementary file 2 — Supplementary Figures. [file 41598_2021_81813_MOESM2_ESM.pdf]

Glucose confers protection to *Escherichia coli* against contact-killing by *Vibrio cholerae*

Cristian V Crisan, Holly Nichols, Sophia Wiesenfeld, Gabi Steinbach, Peter J Yunker, Brian K Hammer

Supplementary Figures

Supplementary Figure 1.

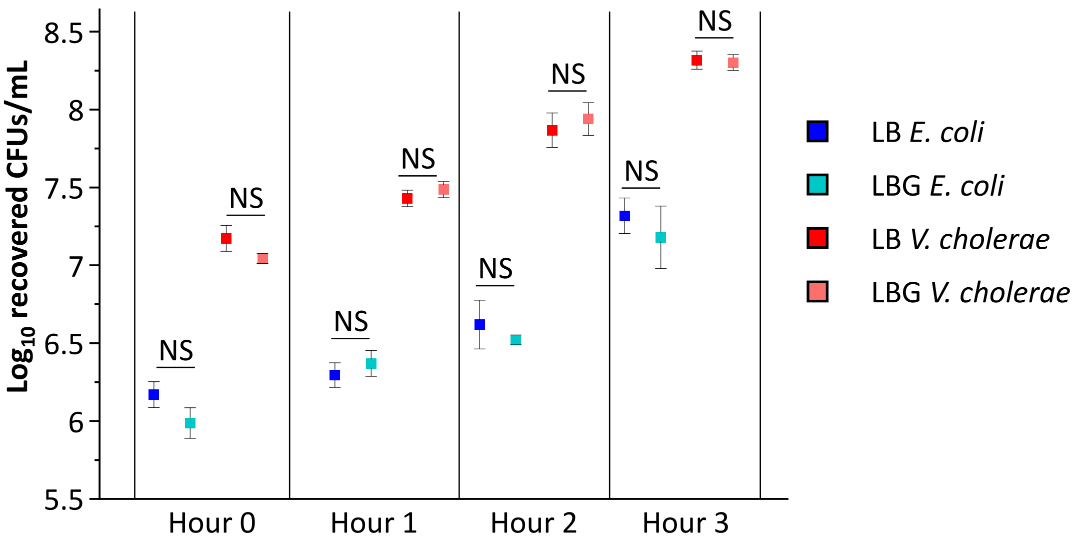

Supplementary Figure 1. Glucose does not significantly alter growth of *E. coli* MG1655 or *V. cholerae* C6706* in monoculture. *E. coli* MG1655 or *V. cholerae* C6706* were grown in monoculture conditions identical to co-culture experiments described in this study. NS not significant. A three-way ANOVA with a post hoc Bonferroni test was used to determine significance. NS – not significant.

Supplementary Figure 2.

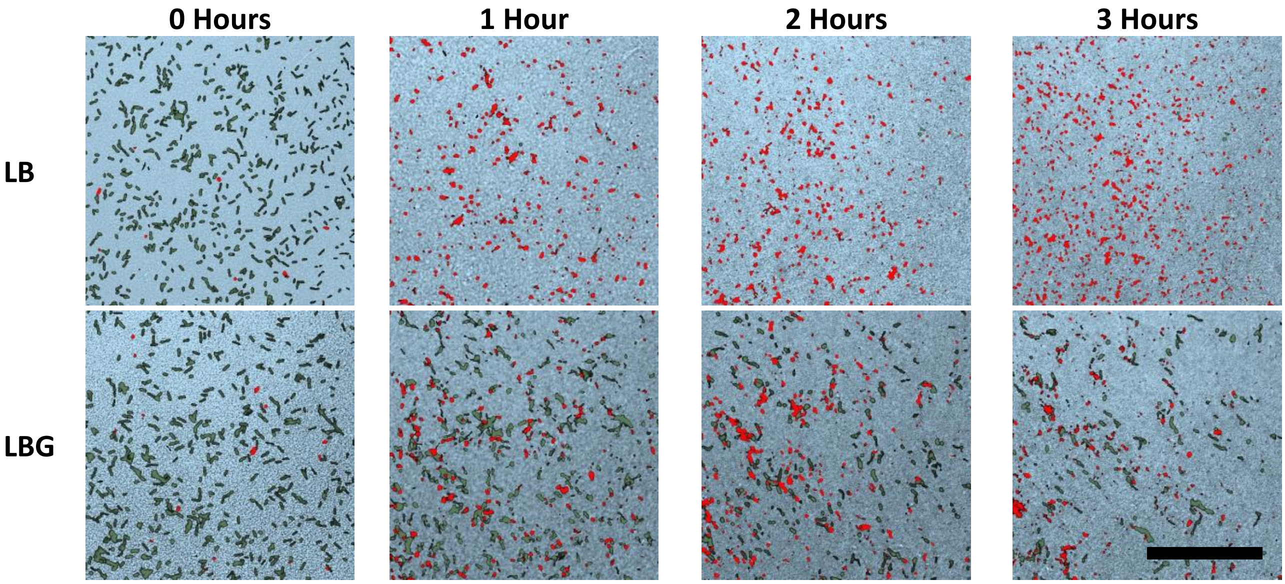

Supplementary Figure 2. *E. coli* cells are permeabilized less on LBG than on LB when competed against killer *V. cholerae* C6706*. Co-cultures of fluorescently labeled green *E. coli* MG1655 cells and unlabeled *V. cholerae* C6706* on LB (top panels) or LBG (bottom panels) were imaged as described in Figure 3A. The same image as Figure 3A is depicted but the propidium iodide signal is also displayed. Scale bar is 50 µm.

Supplementary Figure 3.

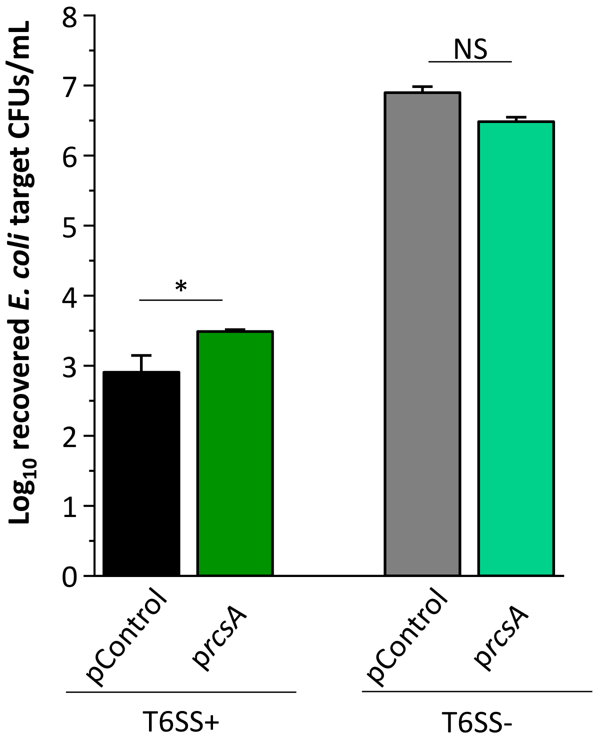

Supplementary Figure 3. Colanic acid confers *E. coli* cell modest protection against *V. cholerae* C6706* T6SS attacks. *E. coli* cells harboring a plasmid control or a plasmid overexpressing *rcsA* were co-cultured with T6SS+ and T6SS- *V. cholerae* on LB. A two-way ANOVA with a post hoc Bonferroni test was used to determine significance. * $p < 0.05$, NS – not significant.

Supplementary Figure 4.

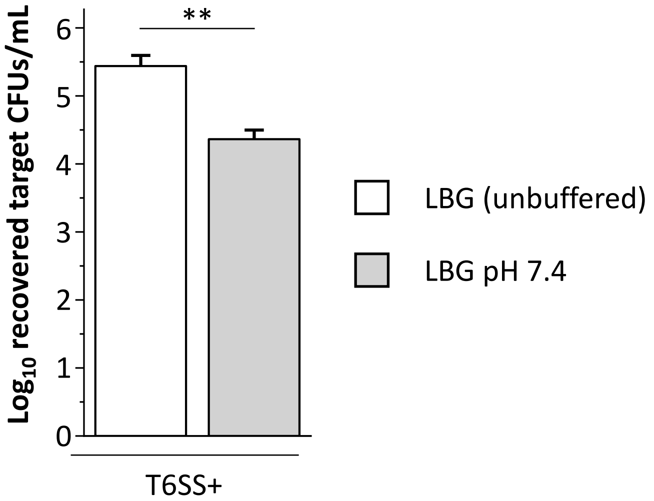

Supplementary Figure 4. Buffered LBG media at a pH of 7.4 increases killing. Target *E. coli* MG1655 and killer *V. cholerae* C6706* were co-cultured on either unbuffered LBG medium or buffered LBG medium at a pH of 7.4. A Welch's T-test was performed to determine significance. ** $p < 0.01$.
